# A single dose of Astragalus saponins adjuvanted inactivated vaccine for pseudorabies virus protected mice against lethal challenge

**DOI:** 10.3389/fvets.2022.1036161

**Published:** 2022-11-21

**Authors:** Chen Chang, Haiyan Wang, Tao Hua, Daohua Zhang, Weibin Hong, Bihua Deng, Bo Tang

**Affiliations:** ^1^Institute of Veterinary Immunology and Engineering, Jiangsu Academy of Agricultural Sciences, Nanjing, China; ^2^National Research Center of Veterinary Bio-product Engineering and Technology, Jiangsu Academy of Agricultural Science, Nanjing, China; ^3^Dongguan Animal Disease Control and Prevention Center, Dongguan, China

**Keywords:** pseudorabies virus (PRV), inactivated vaccine, Astragalus saponins, immunopotentiator, single dose

## Abstract

Pseudorabies (PR) is an important infectious disease of swine that causes enormous economic losses to the swine industry in China. Immunization with vaccines is a routine practice to control this disease. PRV inactivated vaccines usually require a booster vaccination to provide complete immune protection. Therefore, Astragalus saponins (AST) have been added as an immunopotentiator to improve the immune efficacy and reduce the immunization times for the PRV inactivated vaccine. The results in mice have shown that a single dose of AST-adjuvanted PRV inactivated vaccine promoted higher production of gB-specific IgG, IgG1, and IgG2a and neutralizing antibody, secretion of Th1-type (IFN-γ) and Th2-type (IL-4) cytokines, and lymphocyte proliferation than mice immunized without AST. Compared to mice immunized without AST, a single dose of the AST-adjuvanted PRV inactivated vaccine improved the survival percentage of mice and reduced the PRV viral loads in the lungs and brains after lethal challenge. In summary, AST was an effective immunopotentiator to improve the immune efficacy of a single dose PRV inactivated vaccine.

## Introduction

Pseudorabies (PR), also known as Aujeszky's disease (AD), is caused by pseudorabies virus (PRV), which can cause nervous system disorders, respiratory disorders, reproductive failure and fever, and it causes enormous economic losses to the swine industry in many countries (1, 2). PRV is a member of the family Herpesviridae, subfamily Alphaherpesvirinae (3). It was controlled and eradicated in China before 2011 using the Bartha-K 61 attenuated live vaccine. However, since 2011, PR outbreaks in the Bartha-K 61 immunized swine population have caused significant economic losses to the swine industry in China (4). A PRV variant was considered to be the cause of emerging PR since the Bartha-K 61 vaccine did not provide full protection against the prevalent PRV variant in the swine population (2, 5). Compared with the classic PRV strain, the PRV variant had stronger virulence and higher mortality (4). For PRV vaccines, generate high level of neutralizing and gB antibodies could offer effective protection against PRV challenge (6). Bartha-K 61 vaccine generated high neutralizing antibodies against Bartha-K 61, but limited neutralizing antibodies against PRV variant. Therefore, it is necessary to develop more efficient vaccines based on current PRV variants to control PR in China. Currently, gene-deleted modified live vaccines or inactivated vaccines against PRV variants are used to control PR (2, 7–10). A single immunization with modified live vaccines obtained good protective efficacy against PRV challenge (10, 11). However, live vaccines carry the risk of genetic variation. Although inactivated vaccines do not carry this risk, they are not able to provide complete immune protection and usually require a booster vaccination. Therefore, it is necessary to develop new adjuvants to improve the protective efficacy and reduce the immunization times of PRV inactivated vaccines.

Astragalus has been a traditional Chinese herbal medicine for a long period of human history, and it is known for its immunomodulatory, antiviral, and anti-inflammatory functions. Astragalus saponins (AST) are an effective component of Astragalus and have been used to enhance both humoral and cellular immune responses (12). Previous studies have shown that AST significantly enhance the efficacy of vaccination in animals (13, 14). Nilgun Yakubogullari et al. developed an adjuvant system containing AST-VII for the Newcastle disease vaccine, which showed Th1/Th2 balanced antibody and cellular immune responses in a mouse model (15). Thus, these systems could be developed as vaccine adjuvants in viral vaccines as alternatives to saponin-based adjuvants. In this study, AST were added to the PRV inactivated vaccine and used as an immunopotentiator to improve the immune efficacy and develop a single dose of the PRV inactivated vaccine.

## Materials and methods

### Animals

Female ICR mice (6–8 weeks old) were purchased from the Laboratory Animal Center of Yangzhou University (Yangzhou, China). The mice were kept in cages with corncob bedding in a healthy and controlled environment with a stable temperature.

### Cells and virus

Swine testis (ST) cells were purchased from the China Institute of Veterinary Drug Control (Beijing, China) and maintained in Dulbecco's modified Eagle's medium (DMEM, Gibco, Carlsbad, CA, USA) supplemented with 5% calf serum (Gibco), 100 U/ml penicillin (Sigma-Aldrich, St. Louis, MO, USA) and 0.1 mg/ml streptomycin (Sigma-Aldrich, St. Louis, MO, USA). PRV wild strain SQ was isolated from a pig diagnosed with PR in Suqian, Jiangsu Province, China. ST cells were placed in cell flasks at a density of 1 × 10^5^ cells/ml. After 24 h, the culture medium was removed, and fresh DMEM containing 2% calf serum was added and inoculated with PRV strain SQ at a multiplicity of infection (MOI) of 0.001. After 80% of the cells had a marked cytopathic effect (CPE), the cells and medium were harvested and stored at −80°C until use.

### Preparation of vaccines

The inactivated PRV (10^8^ TCID_50_/ml) was prepared by incubating 0.1% formalin (Sigma-Aldrich) at 37°C for 24 h. The PRV vaccine group was created by combining equal parts of the inactivated virus and MONTANIDETMISA 201 adjuvant (SEPPIC, Paris, France). The PRV group was created by combining equal parts of the inactivated virus and PBS. Total AST (Guyan, Shanghai, China) were added to the PRV vaccine or PRV at 100 μg/ml.

### Vaccine efficacy experiment

Seventy-five mice were randomly divided into 5 groups. Mice (*n* = 15/group) were subcutaneously immunized with 100 μl of PRV inactivated antigen (PRV), PRV inactivated antigen with 100 μg/ml AST (PRV/AST), PRV vaccine, or PRV vaccine with 100 μg/ml AST (PRV vaccine/AST). The control group was subcutaneously immunized with PBS. At 28 days post-immunization (dpi), mice from each group (*n* = 5) were euthanized, and the serum was collected for the detection of serum-specific IgG, IgG1, IgG2a and neutralizing antibodies. Splenocytes were isolated for cell proliferation and cytokine assays.

At the same time, the remaining mice (*n* = 10) were intraperitoneally challenged with PRV strain SQ (2500TCID50/mice). The clinical symptoms and body weight changes were observed daily for 14 days. At 14 dpc, the brains or lungs of surviving mice in each group were collected for the quantification of viral loads or histological examination.

### Detection of serological antibodies

A commercial pseudorabies virus gB antibody test kit (Boyan, Wuhan, China) was used to evaluate the serum-specific IgG antibody in the serum. Using the same ELISA procedure, HRP-conjugated goat anti-mouse IgG antibody was replaced by HRP-conjugated goat anti-mouse IgG1 or IgG2a (Thermo Fisher), and then OD 450 values were detected by a microplate reader (BioTek Instruments, USA) to analyze IgG, IgG1 and IgG2a.

### Detection of neutralizing antibodies

Serum samples were collected at 14 and 28 dpi. Briefly, the samples were heat-inactivated at 56°C for 30 min. Two-fold serial dilutions of sera with 100 TCID_50_ PRV SQ strain were incubated for 1 h at 37°C. The virus-serum mixtures were added to confluent monolayers of ST cells in 96-well plates. The plates were incubated for 4–5 days at 37°C in a 5% CO_2_ atmosphere, and the CPE was recorded. Neutralizing antibody titers were calculated based on the Reed-Muench method.

### Lymphocyte proliferation response

Mice from each group were euthanized at 28 dpi, and the spleens were isolated under aseptic conditions and then grated and filtered through a 100-mesh cell sieve. To remove erythrocytes, the splenocytes were treated with ACK Lysis Buffer (Beyotime Biotechnology, Nantong, China) for 5 min at 4°C. Splenocyte suspensions were obtained in RPMI 1640 medium supplemented with 10% FBS containing penicillin (100 IU/ml) and streptomycin (100 μg/ml). After cell counting, 50 μl of splenocytes at a concentration of 1 × 10^6^ cells/ml were cultured in 96-well plates. Then, the splenocytes were stimulated with 50 μl of Con A (5 μg/ml), heat-inactivated PRV antigen (5 × 10^6^ TCID_50_/ml) or RPMI 1640 medium. After incubation for 48 h, 10 μl of CCK8 (Beyotime Biotechnology, Nantong, China) were added to each well, and the incubation was continued for an additional 4 h. The results were expressed as the stimulation index (SI), which was determined based on the following formula: OD values of stimulated wells/OD values of unstimulated wells.

### Measurements of IL-4 and IFN-*γ* production by splenocytes *in vitro*

Splenocytes were restimulated with inactivated PRV antigen (5 × 10^5^TCID_50_) at 37°C for 48 h in a 5% CO_2_ atmosphere. After centrifugation (1500 rpm, 5 min), the quantitative analysis of IL-4 and IFN-γ in the supernatants was performed using commercial ELISA kits (Cat.No.431107, 430807; Biolend, USA) according to the manufacturer's instructions.

### Quantification of viral loads in the tissues

DNA was extracted from the samples using a DNA mini kit (Vazyme, Nanjing, China) according to the manufacturer's instructions. The viral loads in the tissue samples of infected mice were determined using a verified real-time qPCR assay of the PRV gE gene with previously described primers and probes on an ABI7300 Sequence Detection System (v.1.3; Applied Biosystems, Foster City, CA, USA) (9).

### Histological examination

The surviving mice were euthanized and necropsied at 14 dpc. The lungs of these mice were collected and fixed with 4% polyoxymethylene (Beyotime Biotech, Nantong, China); the samples were embedded in wax, cut into transverse sections, and stained with hematoxylin and eosin (H&E).

### Statistical analysis

The experimental data were statistically analyzed using SPSS software (version 20.0; IBM, Armonk, NY, USA). ANOVA with Fisher's LSD *post hoc* test was used for multiple comparisons between groups. The results from the various groups are represented as the mean ± standard deviation (SD), and *p*-values < 0.05 were considered to be statistically significant.

## Results

### Antibody response in mice immunized with AST-adjuvanted inactivated PRV vaccine

**Figure 1:**
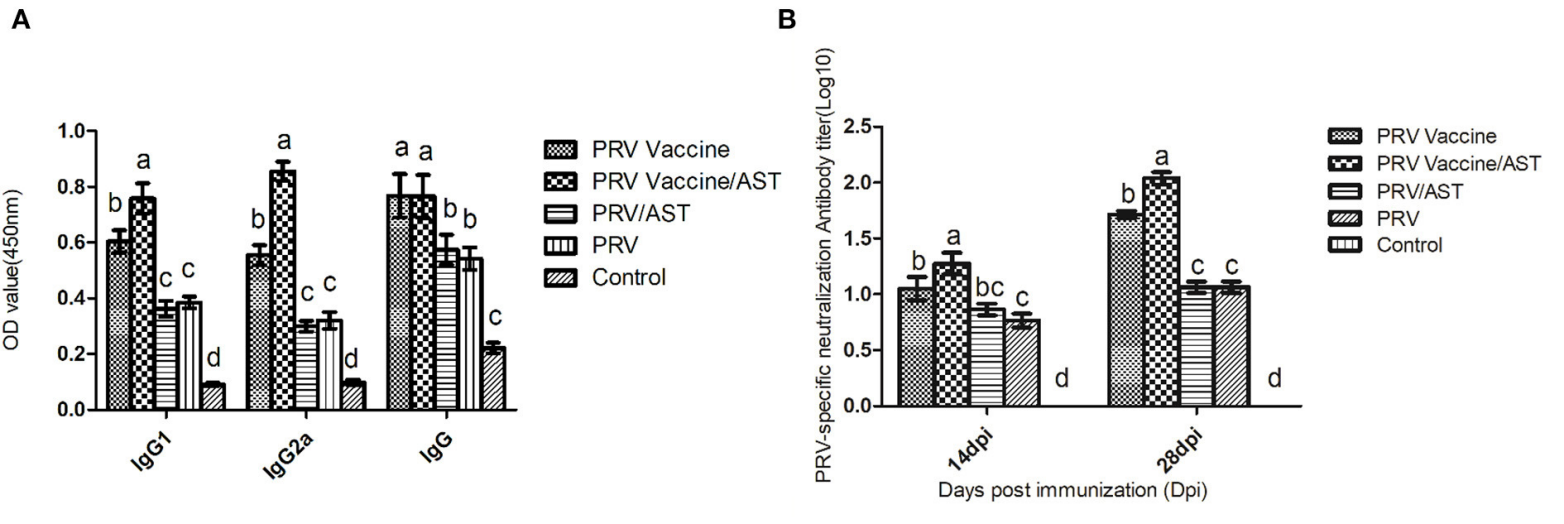
Measurement of the IgG subclasses **(A)** and neutralizing antibody titers **(B)** in the serum at different days post-immunization. At 28 dpi, mice from each group (*n* = 5) with different treatments were euthanized, and serum was collected for the detection of serum-specific IgG and isotypes by ELISA kits **(A)**. At 14 and 28 dpi, the serum was analyzed for PRV-specific neutralizing antibody titers **(B)**. Bars with different letters indicate significant differences (*p* < 0.05).

Mice were immunized with PRV vaccine or PRV with or without AST, and the IgG subclasses and neutralizing antibody titers in the serum were detected to evaluate the adjuvant effect of AST. Figure 1 shows that AST supplementation significantly enhanced the IgG, IgG1, IgG2a and neutralizing antibody responses in the PRV vaccine group (*p* < 0.05). However, AST was not able to enhance the IgG and neutralizing antibody titers in the PRV group (Figures 1A,B).

### Lymphocyte proliferative response in mice immunized with AST-adjuvanted inactivated PRV vaccine

**Figure 2:**
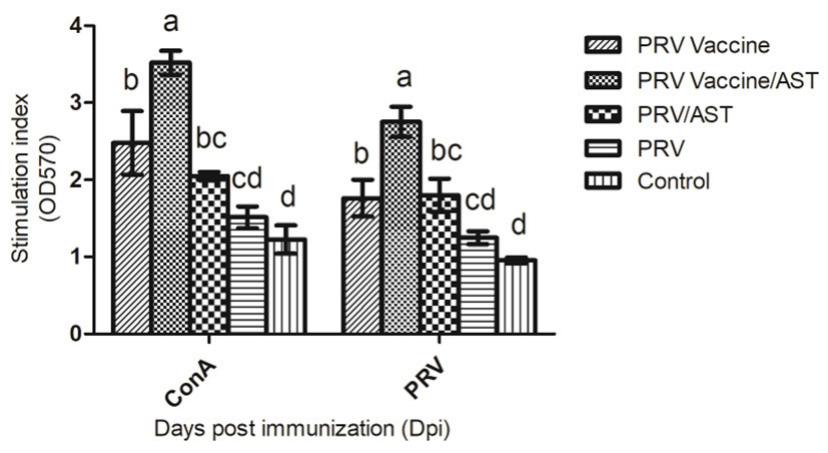
Lymphocyte proliferative response. At 28 dpi, mice from each group (*n* = 5) with different treatments were euthanized, and splenocytes were stimulated with Con A and inactivated PRV antigen for 48 h for analysis of lymphocyte proliferation using the CCK8 method; the results are shown as a stimulation index (SI). Data are expressed as the mean ± SD (*n* = 5). Bars with different letters indicate significant differences (*p* < 0.05).

Lymphocyte proliferation represents the level of cellular immune response. To clarify the effects of AST on the cellular immune responses, the lymphocyte proliferative responses in different groups were detected in this study. Figure 2 indicates that the stimulation index of lymphocyte proliferation in the PRV vaccine/AST group was significantly higher than that in the PRV vaccine alone group (*p* < 0.05) at 28 dpi. However, there were no significant differences between the PRV/AST and PRV groups. These results suggested that AST enhanced the cellular immune response in the PRV vaccine group but not in the PRV group.

### Th1 and Th2-type cytokine release in mice immunized with AST-adjuvanted inactivated vaccine

**Figure 3:**
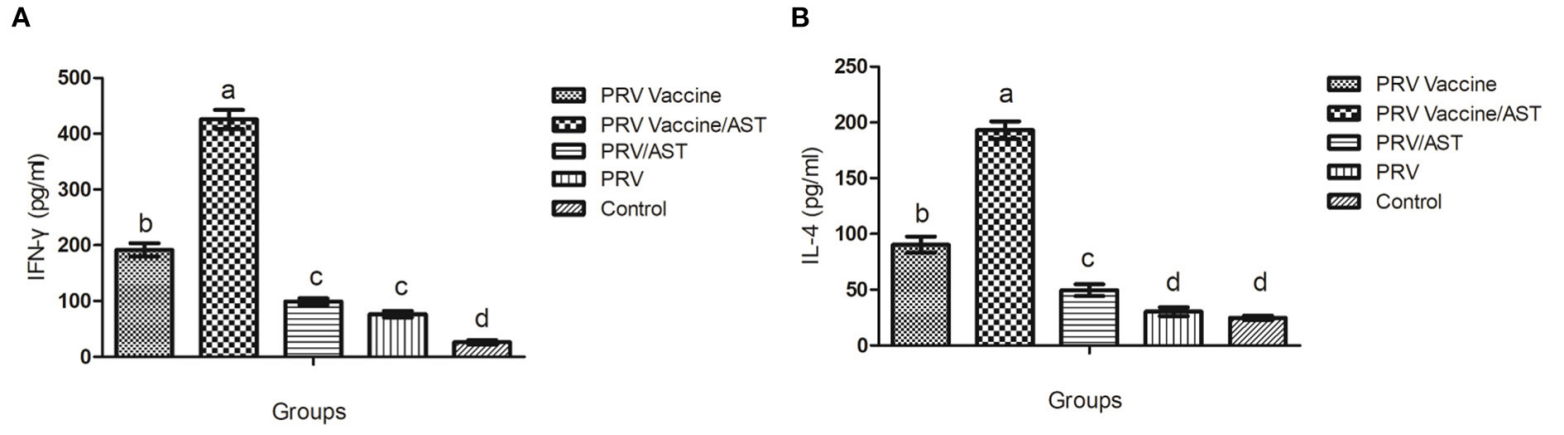
Production of IFN-γ **(A)** and IL-4 **(B)** by splenocytes. At 28 dpi, mice from each group (*n* = 5) with different treatments were euthanized, and the splenocytes were stimulated with inactivated PRV antigen. After 48 h, the concentrations of IL-4 and IFN-γ in the supernatants were tested by commercial ELISA kits. The values represent the mean ± S.D (*n* = 5). Bars with different letters indicate significant differences (*p* < 0.05).

To gain further understanding of the action mode of AST regarding the immune response, cytokine release was determined by evaluating Th1-type cytokine (IFN-γ) and Th2-type cytokine (IL-4) concentrations in the splenocyte supernatant of immunized mice upon stimulation by inactive pseudorabies virus. As shown in Figure 3, the PRV vaccine/AST group generated 2-fold higher concentrations of IFN-γ and IL-4 than the PRV vaccine group and 4-fold higher concentrations than the other groups. There was no difference in the concentration of IFN-γ between the PRV/AST and PRV groups (*p* > 0.05) (Figures 3A,B).

### Protective effect in mice immunized with an AST-adjuvanted inactivated vaccine against PRV challenge

**Figure 4:**
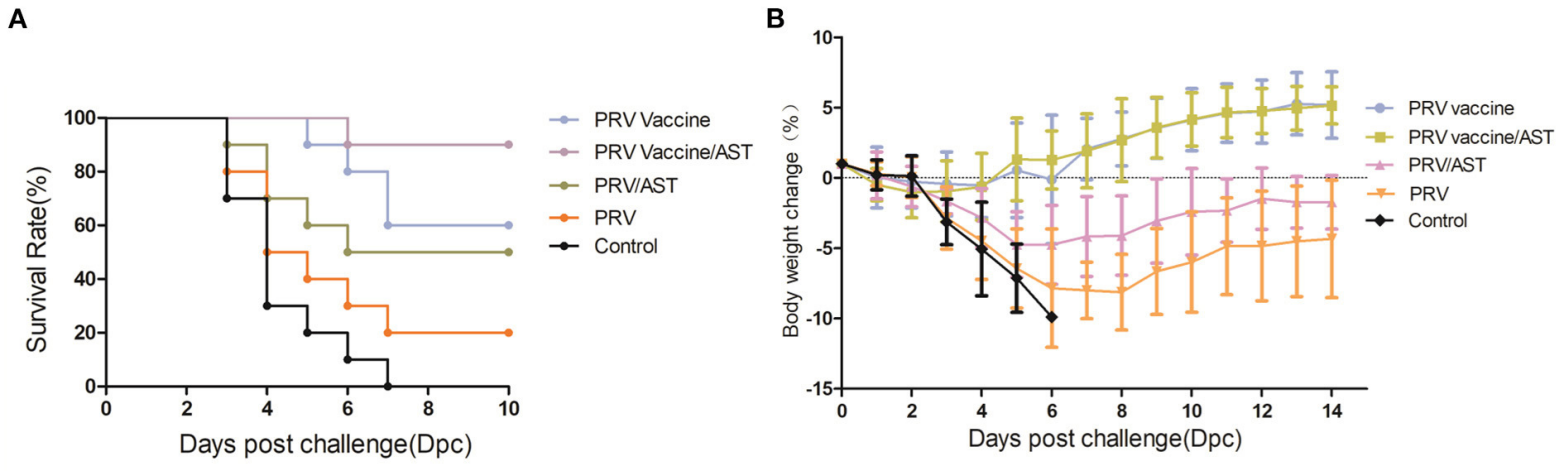
Survival rate and body weight changes of mice after challenge. Mice in different groups were challenged with PRV at 28 dpi, and the survival rate and body weight changes of the mice were analyzed from dpc 0 to dpc 14. Body weight changes of mice in different groups after challenge **(A)**. Survival rates of mice in different groups after challenge **(B)**. Data are the means ± S.D. (*n* = 10).

To evaluate the protective effect of the AST-adjuvanted PRV vaccine, mice were intraperitoneally injected with a lethal dose of PRV at 4 weeks post immunization. The survival rates of these mice after challenge are shown in Figure 4A. Mice in the control group began to show convulsions, ataxia and paralysis as early as 60 h (data not shown) and gradually died at 3 to 7 dpc, while the survival percentages of mice immunized with PRV vaccine/AST, PRV vaccine, PRV/AST and PRV were 90, 60, 50 and 20%, respectively. Therefore, AST was able to enhance the protective effect of the PRV vaccine against PRV challenge.

### Body weight changes in mice immunized with AST-adjuvanted inactivated vaccine after PRV challenge

To evaluate the body weight changes in mice immunized with the AST-adjuvanted inactivated vaccine after PRV challenge, mice were intraperitoneally injected with a lethal dose of PRV at 4 weeks post-immunization and then weighed daily from dpc 0 to 14. The data on body weight changes are shown in Figure 4B. The increases in body weight in the PRV vaccine/AST and PRV vaccine groups were 5.15 ± 1.33 and 5.18 ± 2.36%, respectively, and there was no significant difference between the two groups. In addition, the increase in body weight in the PRV/AST and PRV groups was −1.74 ± 1.91 and −4.34 ± 4.18%, respectively, and reached −9.89% in the control group.

### Quantification of viral loads in the brain and lungs of mice immunized with AST-adjuvanted inactivated vaccine after PRV challenge

**Figure 5:**
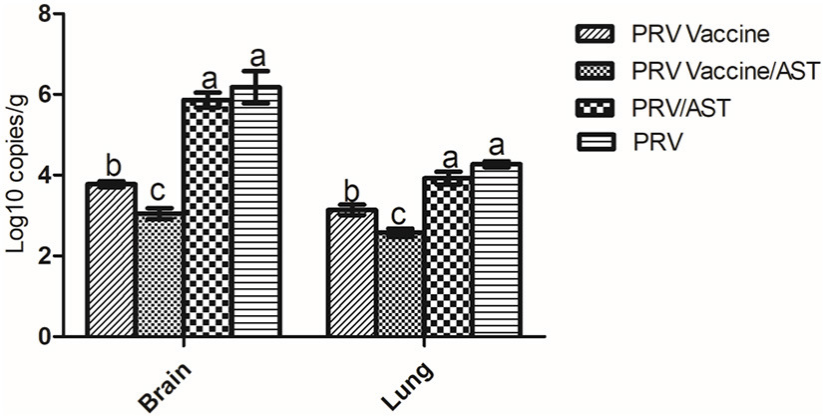
Quantification of viral loads in the brain and lungs of mice after challenge. Mice in different groups were challenged with PRV at 28 dpi, brain and lung samples were collected from each group at 14 dpc, and the viral loads in the brain and lungs were detected by quantitative real-time PCR. Data are expressed as the mean ± S.D.

To evaluate the viral loads in the brains and lungs of mice immunized with the AST-adjuvanted inactivated vaccine after PRV challenge, brain and lung samples were collected from each group, and the viral loads were detected by quantitative real-time PCR (Figure 5). After challenge, the PRV viral loads in the PRV vaccine/AST group were significantly lower than PRV vaccine group (*p* < 0.05). There was no difference between the PRV/AST and PRV groups (*p* > 0.05).

### Histological examination

**Figure 6:**
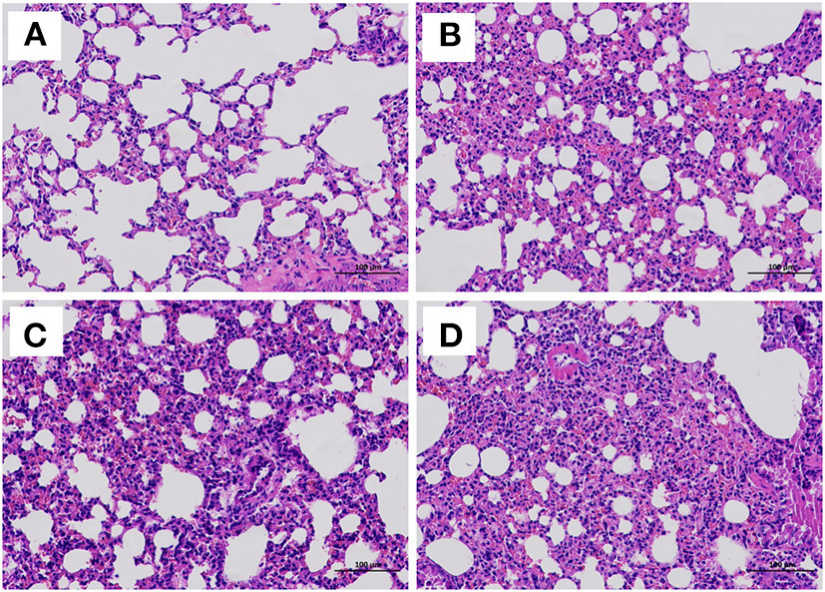
Histological examination of the lungs of mice in different groups at 14 days post-challenge. Mice in different groups were challenged with PRV at 28 dpi, and all surviving mice were euthanized and necropsied. The lungs were collected for histological examination. PRV vaccine/AST group **(A)**, PRV vaccine group **(B)**, PRV/AST **(C)**, PRV group **(D)**.

At the end of the challenge experiment, all live mice were euthanized and necropsied. Tissues were collected for histopathological analysis. The results showed alveolar wall thickening, lymphocyte and neutrophil infiltration and local bleeding in the lungs of PRV/PBS groups (D). The pathological lesions in the PRV vaccine/AST group (A) were significantly milder than those in the PRV vaccine (B), PRV/AST (C) and PRV/PBS groups (D) (Figures 6A–D).

## Discussion

PR outbreaks have occurred in the Bartha-K 61 immunized swine population since 2011. The genome analysis showed that PRV variants with stronger virulence and higher mortality had substitutions, insertions and deletions compared to previous classic strains (2). Therefore, traditional attenuated pseudorabies vaccines cannot completely prevent pigs from PRV infection (9). Currently, some means are used to control PR, including immunization with gene-deleted modified live vaccines or inactivated vaccines against PRV variants. Live vaccines, which generally induce long-lasting immunity, carry a risk of insufficient attenuation and genetic instability. Inactivated vaccines are less efficient than attenuated vaccines and require repeated doses (16). In addition, repeat vaccination in pigs is time-consuming and labor-intensive and increases the risk of disease transmission. Thus, it is necessary to develop a safe and efficient PRV inactivated vaccine with single-dose. In this study, we found that a single dose of AST-adjuvanted inactivated PRV vaccine provided sufficient immune protection and protected mice against lethal challenge. It was safe and efficient, which would reduce the dose and frequency of immunizations, and save the labor in the pig farm.

Traditional Chinese medicine has been widely used for immune activators in vaccine formulation, and it can provide a faster and stronger immune response (17). Saponin-based adjuvants, such as AST, have the ability to stimulate the cell-mediated immune system and enhance antibody production, and only a low dose is needed for adjuvant activity (18). Astragalus is able to increase the transformation of macrophages and T cells, interferon, and phagocytosis (18). In addition, a combination of adjuvants usually yield better immune responses than either adjuvant component alone; reduce dose of individual adjuvant component and frequency of immunizations; and may induce humoral immune response and cellular immune response (19). It was proven that the involvement of AST in oil adjuvant production was beneficial in improving the immune response (20).Therefore, AST were used as an immunopotentiator to improve the efficacy of a single dose of PRV inactivated vaccine in this study.

Pigs have been confirmed as the primary host of PRV. Besides the pig, PRV has a wide spectrum of hosts, including mice, cattle, sheep, dog, cat et al. (21, 22). Therefore, mice model is usually used to evaluate the efficacy of PRV vaccine (23, 24). In this study, we found that AST increased the survival rate of mice from 60% in the PRV vaccine group to 90% in the PRV vaccine/AST group after lethal PRV challenge, which was equivalent to that of the PRV vaccine after repeated doses (data not shown). In addition, AST-adjuvanted PRV vaccine significantly reduced the amount of virus and number of tissue lesions in the lungs. These results indicated that a single dose of the AST-adjuvanted PRV inactivated vaccine protected mice against lethal challenge.

gB is a main component of the PRV envelope, which induces protective immunity. Antibodies produced in mice and pigs to purify gB and antibodies generated in mice to recombinant gB were able to neutralize PRV *in vitro* (25, 26). In this study, AST in the PRV vaccine/AST group significantly improved PRV gB-specific antibody and neutralizing antibody production compared to the other groups.

Lymphocyte proliferation is an important indicator of the immune response and reflects the level of cellular immunity (27). To clarify the effects of AST on cellular immunity, the ability of Con A or PRV antigen to stimulate the proliferation of splenocytes was investigated. PRV vaccine containing AST significantly increased the proliferation of splenocytes compared with PRV vaccine alone at 28 dpi, proving that AST can promote T-cell immune responses. In addition, IFN-γ is one of the crucial cytokines in antiviral immunity due to its immunoregulatory properties, such as the activation of cytotoxic T lymphocytes and natural killer cells and the recruitment of macrophages to the injection site (28). IFN-γ is related to the Th1 immune response, which is effective for protection against intracellular infections (29, 30). IL-4 is related to the Th2 immune response, which is effective for protection against extracellular infections, helping B cells to produce antibodies (9). This study also found that AST enhanced the production of IFN-γ and IL-4, suggesting that both Th1 and Th2 subset cells were activated by AST.

Among these results, we also found that AST alone was not able to effectively enhance the PRV antibody and IFN-γ production, and to reduce the viral loads of the brain and lungs in the PRV/AST group, which did not contain a mineral 201 adjuvant. The mineral oil component in the 201 adjuvant mainly played a slow release role, which was conducive to the slow release of antigen or AST, delaying the degradation of antigen *in vivo*, so as to stimulate the immune system more effectively. Therefore, AST enhanced the immune response in the PRV vaccine group but not in the PRV group.

## Conclusion

In conclusion, we found that a single dose of AST-adjuvanted inactivated PRV vaccine significantly improved cellular and humoral immune responses and protected mice against lethal challenge. These results indicated that AST could be used as an immunopotentiator for a PRV-inactive vaccine, which could provide adequate immune protection with a single dose. Further studies are also needed to clarify the efficacy of AST in pigs for the development of a single-dose inactivated PRV vaccine.

## Data availability statement

The original contributions presented in the study are included in the article/supplementary material, further inquiries can be directed to the corresponding authors.

## Ethics statement

The animal study was reviewed and approved by Jiangsu Academy of Agricultural Sciences Experimental Animal Ethics Committee.

## Author contributions

Conceived and designed the experiments: CC, HW, BT, and BD. Performed the experiments: CC, TH, and WH. Analyzed the data: CC, HW, DZ, and BT. Contributed reagents, materials, and analysis tools: BD and BT. Wrote and editing the paper: CC, HW, and BT. All authors contributed to the article and approved the submitted version.

## Funding

This work was supported by exploring and overturning of Jiangsu Academy of Agricultural Sciences [ZX(21)1219] and Jiangsu province Natural Sciences Foundation (BK20221431).

## Conflict of interest

The authors declare that the research was conducted in the absence of any commercial or financial relationships that could be construed as a potential conflict of interest.

## Publisher's note

All claims expressed in this article are solely those of the authors and do not necessarily represent those of their affiliated organizations, or those of the publisher, the editors and the reviewers. Any product that may be evaluated in this article, or claim that may be made by its manufacturer, is not guaranteed or endorsed by the publisher.
